# Supplementary material for: The incidence and severity of COVID-19 in adult professional soccer players in Russia
Source: PLoS One. 2022 Jun 6;17(6):e0265019. doi: 10.1371/journal.pone.0265019 (PMC9170087; doi:10.1371/journal.pone.0265019)
Supplement: Attachment — Submitted filename: PONE-D-21-35577_reviewer 11.pdf [file pone.0265019.s002.pdf]

PLOS ONE

The Incidence and Severity of COVID-19 in Adult Professional Soccer Players

--Manuscript Draft--

**Manuscript Number:**	PONE-D-21-35577
**Article Type:**	Research Article
**Full Title:**	The Incidence and Severity of COVID-19 in Adult Professional Soccer Players
**Short Title:**	COVID-19 in Soccer Players
**Corresponding Author:**	Zbigniew Waśkiewicz<br>Jerzy Kukuczka Academy of Physical Education In Katowice: Akademia Wychowania Fizycznego imienia Jerzego Kukuczki w Katowicach<br>Katowice, POLAND
**Keywords:**	COVID-19; elite professional soccer; infectious disease
**Abstract:**	At present, there are little data on incidence and clinical course of COVID-19 among professional soccer players, and the studies examining putative complications of COVID-19 infections are probabilistic. Thus, examining the incidence of COVID-19 and various aspects of its clinical course in a group of adult professional soccer players would be of great practical interest. The incidence, clinical course, and severity of COVID-19 infection, as well as the duration of treatment and return to play was studied based on a survey of team physicians and medical records assessment in the group of adult professional soccer players representing the clubs of the Russian Premier-League (RPL) during the period of championship resumption from 01.04.2020 until
**Order of Authors:**	Zbigniew Waśkiewicz
	Eduard Bezuglov
	Artemii Lazarev
	Evgeniy Achkasov
	Vladimir Khaitin
	Larisa
	Romanova67
	Mikhail Butovskiy
	Vladimir Khokhlov
	Maxim Tsyplenko
	Alexander
	Linskiy11
	Petr Chetverikov
	Magomedtagir Sugaipov
	Arseniy Petrov
**Additional Information:**	
**Question**	**Response**
**Financial Disclosure**<br><br>Enter a financial disclosure statement that describes the sources of funding for the work included in this submission. Review the submission guidelines for detailed requirements. View published research	The author(s) received no specific funding for this work.

Powered by Editorial Manager® and ProduXion Manager® from Aries Systems Corporation

articles from *PLOS ONE* for specific examples.<br><br>This statement is required for submission and **will appear in the published article** if the submission is accepted. Please make sure it is accurate.<br><br>**Unfunded studies**<br>Enter: *The author(s) received no specific funding for this work.*<br><br>**Funded studies**<br>Enter a statement with the following details:<br>• Initials of the authors who received each award<br>• Grant numbers awarded to each author<br>• The full name of each funder<br>• URL of each funder website<br>• Did the sponsors or funders play any role in the study design, data collection and analysis, decision to publish, or preparation of the manuscript?<br>• **NO** - Include this sentence at the end of your statement: *The funders had no role in study design, data collection and analysis, decision to publish, or preparation of the manuscript.*<br>• **YES** - Specify the role(s) played.<br><br>* typeset	
**Competing Interests**<br><br>Use the instructions below to enter a competing interest statement for this submission. On behalf of all authors, disclose any competing interests that could be perceived to bias this work—acknowledging all financial support and any other relevant financial or non-financial competing interests.<br><br>This statement is required for submission and **will appear in the published article** if the submission is accepted. Please make sure it is accurate and that any funding sources listed in your Funding Information later in the submission form are also declared in your Financial Disclosure statement.	The authors have declared that no competing interests exist.

View published research articles from *PLOS ONE* for specific examples.<br><br>**NO authors have competing interests**<br><br>Enter: *The authors have declared that no competing interests exist.*<br><br>**Authors with competing interests**<br><br>Enter competing interest details beginning with this statement:<br><br>*I have read the journal's policy and the authors of this manuscript have the following competing interests: [insert competing interests here]*<br><br>* typeset	
**Ethics Statement**<br><br>Enter an ethics statement for this submission. This statement is required if the study involved:<br><br>• Human participants<br>• Human specimens or tissue<br>• Vertebrate animals or cephalopods<br>• Vertebrate embryos or tissues<br>• Field research<br><br>Write "N/A" if the submission does not require an ethics statement.<br><br>General guidance is provided below. Consult the submission guidelines for detailed instructions. **Make sure that all information entered here is included in the Methods section of the manuscript.**	The study was approved by the local ethics committee (Sechenov University, protocol № 30-20 from 21.10.2020)

**Format for specific study types**<br><br>**Human Subject Research (involving human participants and/or tissue)**<br>• Give the name of the institutional review board or ethics committee that approved the study<br>• Include the approval number and/or a statement indicating approval of this research<br>• Indicate the form of consent obtained (written/oral) or the reason that consent was not obtained (e.g. the data were analyzed anonymously)<br><br>**Animal Research (involving vertebrate animals, embryos or tissues)**<br>• Provide the name of the Institutional Animal Care and Use Committee (IACUC) or other relevant ethics board that reviewed the study protocol, and indicate whether they approved this research or granted a formal waiver of ethical approval<br>• Include an approval number if one was obtained<br>• If the study involved *non-human primates*, add *additional details* about animal welfare and steps taken to ameliorate suffering<br>• If anesthesia, euthanasia, or any kind of animal sacrifice is part of the study, include briefly which substances and/or methods were applied<br><br>**Field Research**<br><br>Include the following details if this study involves the collection of plant, animal, or other materials from a natural setting:<br>• Field permit number<br>• Name of the institution or relevant body that granted permission	
**Data Availability**<br><br>Authors are required to make all data underlying the findings described fully available, without restriction, and from the time of publication. PLOS allows rare exceptions to address legal and ethical concerns. See the PLOS Data Policy and FAQ for detailed information.	Yes - all data are fully available without restriction

A Data Availability Statement describing where the data can be found is required at submission. Your answers to this question constitute the Data Availability Statement and **will be published in the article**, if accepted.<br><br>**Important:** Stating 'data available on request from the author' is not sufficient. If your data are only available upon request, select 'No' for the first question and explain your exceptional situation in the text box.<br><br>Do the authors confirm that all data underlying the findings described in their manuscript are fully available without restriction?	
**Describe where the data may be found in full sentences. If you are copying our sample text, replace any instances of XXX with the appropriate details.**<br><br>• If the data are **held or will be held in a public repository**, include URLs, accession numbers or DOIs. If this information will only be available after acceptance, indicate this by ticking the box below. For example: *All XXX files are available from the XXX database (accession number(s) XXX, XXX.*).<br>• If the data are all contained **within the manuscript and/or Supporting Information files**, enter the following: *All relevant data are within the manuscript and its Supporting Information files.*<br>• If neither of these applies but you are able to provide **details of access elsewhere**, with or without limitations, please do so. For example:<br><br>*Data cannot be shared publicly because of [XXX]. Data are available from the XXX Institutional Data Access / Ethics Committee (contact via XXX) for researchers who meet the criteria for access to confidential data.*<br><br>*The data underlying the results presented in the study are available from (include the name of the third party*	The datasets generated and analyzed during the current study are available from the corresponding author on reasonable request.

and contact information or URL).<br>• This text is appropriate if the data are owned by a third party and authors do not have permission to share the data.<br><br>* typeset	
Additional data availability information:	

Powered by Editorial Manager® and ProduXion Manager® from Aries Systems Corporation

Manuscript

Click here to access/download;Manuscript;The Incidence and Severity of COVID-19 .pdf

The Incidence and Severity of COVID-19 in Adult Professional Soccer Players

Eduard Bezuglov[1,2,3], Artemii Lazarev[1,3,4], Evgeniy Achkasov[1], Vladimir Khaitin[5], Larisa Romanova[6,7], Mikhail Butovskiy[8], Vladimir Khokhlov[9], Maxim Tsyplenko[10], Alexander Linskiy[11], Petr Chetverikov[12], Magomedtagir Sugaipov[13], Arseniy Petrov[14], Oleg Talibov[3,15], Zbigniew Waśkiewicz[1,16]

[1]Department of Sport Medicine and Medical Rehabilitation, Sechenov First Moscow State Medical University (Sechenov University), Moscow, Russia; (E. Achkasov 2215.g23@rambler.ru)

[2]Federal Research and Clinical Center of Sports Medicine and Rehabilitation of Federal Medical Biological Agency, Moscow, Russia;

[3]High Performance Sport Laboratory, Moscow Witte University, Moscow, Russia; (E. Bezuglov e.n.bezuglov@gmail.com)

[4]A.I. Burnazyan Federal Medical and Biophysical Center, Moscow, Russia;

[5]FC Zenit, St. Petersburg, Russia; (V. Khaitin khaitinvladimir@gmail.com);

[6]Head center for hygiene and epidemiology of the Federal Medical Biological Agency, Moscow, Russian Federation;

[7]Academy of Postgraduate Education under the Federal State Budgetary Unit “Federal Scientific and Clinical Center for Specialized Medical Assistance and Medical Technologies of the Federal Medical Biological Agency”, Moscow, Russian Federation; (L. Romanova romanova.larisa2009@yandex.ru)

[8]FC Rubin, Kazan, Russia; (M. Butovskiy drmike81@inbox.ru)

[9]FC Rostov, Rostov-on-Don, Russia; (V. Khokhlov adim@list.ru)

[10]FC Tambov, Tambov, Russia; (M. Tsyplenko info@smartrecovery.su)

[11]FC Sochi, Sochi, Russia; (A. Linskiy linskiy03@yandex.ru)

[12]FC Orenburg, Orenburg, Russia; (P. Chetverikov ontario74@yandex.ru)

[13]FC Akhmat, Grozniy, Russia; (M. Sugaipov s.m-tagir.h@mail.ru)

[14]University of Göttingen, Göttingen, Germany; (A. Petrov petroffsenya@gmail.com)

[15]Moscow State University of Medicine and Dentistry, Moscow, Russia; (O. Talibov olegtalibov@gmail.com)

16, Institute of Sport Sciences, Jerzy Kukuczka Academy of Physical Education, Katowice, Poland

*Corresponding author: Zbigniew Waśkiewicz, z.waskiewicz@awf.katowice.pl

Abstract

At present, there are little data on incidence and clinical course of COVID-19 among professional soccer players, and the studies examining putative complications of COVID-19 infections are probabilistic. Thus, examining the incidence of COVID-19 and various aspects of its clinical course in a group of adult professional soccer players would be of great practical interest. The incidence, clinical course, and severity of COVID-19 infection, as well as the duration of treatment and return to play was studied based on a survey of team physicians and medical records assessment in the group of adult professional soccer players representing the clubs of the Russian Premier-League (RPL) during the period of championship resumption from 01.04.2020 until 20.09.2020. COVID-19 infection was detected in 103 soccer players during COVID-19 screening. This number comprises 14.5% of all soccer players who were on the rosters of RPL soccer teams and were subjects to regular COVID-19 testing.

The asymptomatic course was observed in 43.7% of cases (n=45). These players were isolated and their clinical condition was monitored closely. Clinical symptoms were observed in 56.3% of cases (n=58), the most common symptoms were fatigue, headache, fever, and anosmia. COVID-19 infection was commonly diagnosed among adult professional soccer players continuously residing in Russia. However, the majority of cases had a mild course and did not impair return to regular exercise. Only two players were hospitalized with lung lesions with the subsequent return to regular sports.

Keywords: COVID-19; elite professional soccer; infectious disease

Introduction

There have been very few studies to date that have investigated the clinical course of COVID-19 infection and its impact on the performance of soccer players [1,2]. COVID-19 complications such as pulmonary fibrosis, cardiac and hepatic consequences are actively studied [3]. Until now, the course of COVID-19 infection and its impact on athletic performance has not been studied in professional athletes. Nearly all soccer events had been cancelled in March 2020 due to fast spreading of the global COVID-19 pandemic. The majority of soccer players had to stop training [4–6]. During May-June 2020 sports events were resumed in several countries. However, the events took place without fans in most cases.

In all cases when the events were resumed, the organizing sports leagues developed strict prevention and control measures to minimize risk of infection for the participants [1,2,7]. The key elements of these measures are as follows: close monitoring of the infection rates by PCR-tests; individual face masks and gloves wearing; surface and skin disinfection; adherence to social distancing guidelines. Nevertheless, media reports covering new infection cases among soccer players illustrate that as efficient as they are, these prevention measures cannot completely rule out possibility of COVID-19 spreading in such a numerous population as soccer players.

However, there is presently limited data on incidence and clinical course among professional soccer players, and the studies examining putative complications of COVID-19 infections are probabilistic. Thus, examining various aspects of COVID-19 incidence and clinical course in a group of adult professional soccer players would be of great practical importance

Materials and methods

Incidence, clinical course, and severity of COVID-19 infection, as well as duration of treatment and recovery before return to play were studied based on a survey of team physicians and medical records assessment in the group of adult professional soccer players representing the Russian Premier-League (RPL) clubs during the period of championship resumption from 01.04.2020 until 20.09.2020.

The data of 710 soccer players who were on the rosters of 16 RPL clubs and 2 National Football League teams (second-tier soccer league in Russia) were included in the analysis. The study included players who were in the teams' applications for the season and who were regularly tested in accordance with the existing national sanitary regulations.

According to the RPL COVID-19 regulation, each player registered for a soccer match has to undergo COVID-19 screening by submitting a throat and nostrils swab for a PCR test 3 days before the first play, and once weekly thereafter [6]. Swabs were performed only by specialists from laboratories certified by the Federal Service for surveillance on consumer rights protection “Rospotrebnadzor” - which is a central Russian governmental entity responsible for sanitary and epidemiological surveillance. Teams used different laboratories. However, all laboratories had state accreditation. Without this test, the special QR-code to access the event would not be issued. All positive test results were automatically submitted to a centralized database and then revised in special reference-laboratories. Test results were available within 24-48 hours in the majority of cases. According to the Russian quarantine rules, all individuals tested positive for COVID-19 have to be isolated for 14 days regardless of their clinical symptoms. The quarantine can be lifted only after receiving 2 negative PCR-test results performed within at least 24 hours.

Medical records of athletes diagnosed with COVID-19 by a PCR test were studied. Disease course (symptomatic/asymptomatic), the frequency of pulmonary involvement, and the severity of pulmonary lesions were assessed. The prevalence of distinct clinical findings, the therapeutic approaches, the duration of treatment and recovery before return to play were included in the analysis. The team physicians were interviewed by telephone using a questionnaire compiled in

advance. All necessary information was obtained from the team physicians and in some situations from the athletes themselves. For the monitor of asymptomatic players during quarantine, they themselves measured their temperature and oxygen saturation on a daily basis, and also had to report the onset of any of the symptoms that appeared: cough, shortness of breath, loss of smell or taste, sore throat and headache. Most often this was happened when they were outside the team (at home or in the room at the training base). In such situations, the player was immediately advised to isolate himself.

All the medical records were assessed by an independent clinical pharmacologist. All radiographic findings were initially assessed by radiologists experienced in characterizing lung lesions. All players underwent regular testing in accordance with the adopted sanitary regulations, which made it possible to identify and timely isolate initially asymptomatic carriers. Such measures were not taken in Russia in relation to representatives of the general population at that time.

The database was created with Microsoft Excel software, statistical analysis was performed utilizing the IBM SPSS 23.0 (Armonk, USA). Continuous data were tested for normality of distribution with Kolmogorov-Smirnov test. Normally distributed data were described with Mean (M) and standard deviation (SD). Median (Me) and quartiles were used in case of abnormal distribution). Percentage and absolute numbers were provided for categorical data. Mann-Whitney U-test was performed to compare the duration of treatment and recovery before return to play between athletes with and without pulmonary lesions. Spearman's correlation was used for non-normal distributed data. Results were considered statistically significant at $p < 0.05$.

The study was approved by the local ethics committee (Sechenov University, protocol № 30-20 from 21.10.2020). Players provided written informed consent to participate in the survey. It was explained to them that the use of their medical documentation only served for scientific purposes and their personal data would be protected.

Results

COVID-19 infection was detected in 103 soccer players in the course of COVID-19 screening (average age: 25,1 ± 4,3 years, height: 183,7 ± 6,3 cm, weight: 76,6 ± 7,0 kg, BMI: 22,7 ± 1,4). This number comprises 14.5% of all soccer players who were on the rosters and underwent regular PCR testing. Out of the 103 infected players defensive midfielders were most frequently infected (n=42; 40.8%), followed by defenders (n=30; 29.1%), strikers (n=19; 18.4%) and goalkeepers (n=12; 11.7%). The asymptomatic course was observed in 43.7% of cases (n=45). These players were isolated and their clinical condition was monitored closely. Clinical symptoms were observed in 56.3% of cases (n=58), the most common symptoms being fatigue, headache, fever, and anosmia (Table 1).

Insert Table 1 near here

In all COVID19 PCR-positive cases lung CT scan was performed. Pulmonary lesions were detected in 36.2% (n=21) of symptomatic soccer players. and in 23.3% (n=24) of players with positive test results (in 3 cases, pulmonary lesions were revealed in asymptomatic players, .In asymptomatic cases "frosted glass opacities" extended less than in 10% of lung. There were no fundamental differences in the structure of radiological changes in these players in comparison with the radiological data of football players with clinical symptoms. All three asymptomatic players underwent CT because of their own willing which undoubtedly did not correspond to the existing clinical recommendations.

In all 24 cases pulmonary lesion size was derived from CT images. Less than 10% of lung parenchyma were involved in 70.9% (n=17) of cases, 11-20% in 16.6% (n=4), and 20-29% in 8.3% (n=2) of cases. Lesions involving more than 30% of lung parenchyma were detected only once (4.2%; n=1). Two players (strikers 20 and 36 years old) were hospitalized with lung lesions (26% and 32%). They did not use oxygen therapy during hospital staying. Pulmonary lesions were most commonly associated with the following symptoms: fatigue (76.2%, n=16), headache

(71.4%, n=15), t ≥ 38 °C (52.4%, n=11). Medical records of 59.2% of players (n=61) were available for assessment. Players were clustered by the drug received (Table 2).

Insert Table 2 near here

The average treatment duration of symptomatic soccer players without pulmonary manifestations of the disease was 14,4 ± 4,8 days, and 17,4 ± 3,5 days in those with pulmonary lesions (p=0.0083). Pulmonary lesions were not identified as a risk factor contributing to a longer duration of treatment, which were defined as the time for the disappearance of all symptoms (logistic regression, p = 0.09) or delaying the resumption of training as a regular player in the respective soccer team (logistic regression, p = 0.17).

Significant difference in treatment duration of players with and without pulmonary lesions was registered (p = 0.022, Mann-Whitney), and no significant difference has been observed in duration of recovery before return to play (period of return to regular training activity, which was defined as the period from the moment of diagnosis of the disease to the start of training specific to the sport) (p = 0.29, Mann-Whitney). Treatment duration was significantly longer in the players with pulmonary lesions. There were no complications required oxygen support or pulmonary ventilation. Low-flow oxygen support was not used in any case.

After recovery before return to training all players underwent thorough medical examination which is mandatory in Russia according to the Ministry of Health special order., Admission of any of the football players to participate in competitions and regular training occurs only after passing mandatory medical examination, which should be performed twice a year. This examination is carried out only in clinics that have a special license. According to the Russian Premier League sanitary regulations , all football players diagnosed with any form of COVID-19 were required to undergo a similar examination, even if they had previously passed it. This examination includes physical examination, clinical blood test and extended biochemistry panel, urine test, ECG (rest and stress-test), cardiac and internal organs ultrasound, spirography, chest X-ray examination, stress-test with gas analysis. During this medical examination no cardiovascular,

pulmonary, and hematological abnormalities were detected in players who recovered from COVID-19 infection.

The average duration of recovery before return to play was 18.0 ± 5.0 days (median±IQR). The recovery period lasted 18.0 ± 8.0 days (non-normal distribution) in those who suffered COVID pneumonia and 18.0 ± 5.0 days (non-normal distribution) in those who had no pulmonary manifestations. No significant correlation in COVID-pneumonia and the duration of recovery period was detected (Mann-Whitney, $p = 0.32$). The disease severity was assessed by the number of clinical findings. In all cases, the COVID-19 disease was mild and the treatment algorithm in every case was determined individually. Distribution of age and clinical findings is non-normal; thus, Spearman's rank correlation coefficient was utilized. There were no significant and strong correlation between the age and clinical findings ($p = 0.1$; $R = 0.18$). Almost all soccer players who had symptomatic COVID-19 disease had already participated in soccer events to the moment of study recruitment.

Discussion

We have demonstrated the predominance of asymptomatic forms of the COVID-19 infection among professional soccer players continuously residing in Russia. If symptoms occurred the course of the disease was mild in all cases. There were no severe complications. COVID19did not impair return to training after convalescence. This is the first study to investigate the disease progression of COVID-19 in professional soccer players with a focus on clinical and radiological findings.

Grant et al. performed a systematic review and a meta-analysis of 148 scientific papers, including a total of 24410 adult patients with COVID-19 disease (age >16 years, average age 49 years) from 9 countries. The most common symptoms described were as follows: fever – 78%, cough – 57%, fatigue – 31%. Headache was observed in 13% of cases, hyposmia – 25%, and myalgia - 17%. The most common symptoms in our study were as follows: fatigue (72.4%),

headache (65.5%) and fever > 37.5°C (44.8%). Anosmia was observed in 41.3% of cases. Only 56.3% of soccer players had symptomatic disease, 43.7% were asymptomatic. In contrast to our study, Grant et al. assessed all symptoms in patients with a positive test result. Thus, the symptomatic course of the disease was less commonly observed in our study, which is most probably due to the younger age and excellent health of the soccer players [8].

Bernheim et al. performed a retrospective study of 121 symptomatic COVID-19 patients from China (average age 45±15.6 years). Pulmonary lesions were observed in 78% of patients. In our study, only 20.4% of soccer players had pulmonary manifestations, which is probably due to timely diagnosis and early treatment start [9]. Meyer et al. [7]demonstrated a relatively low incidence of SARS-CoV-2 infections among German soccer players. During the study period from May to July 2020 (9 weeks), 8 players were tested positive in the first PCR-testing round before the resumption of training sessions, 2 players were tested positive in the third round of PCR-testing, and 22 players remained seropositive throughout the whole season. The numbers reported in this study are much lower than ours. The differences might be explained by several factors.

When club training and competitive matches in Russia were resumed, the fans were allowed to enter the stadium, which facilitates the spread of infection. According to the sanitary regulations, players could not intersect with the spectators in any way, since they immediately got out of the bus into the "clean" zone, which was the under-stands room and the field and the locker rooms. All people (except players) who were in the "clean zone" could get there only by passing a negative PCR test within 72 hours before the start of the match. In contrast to Russia, viewers were not allowed at Bundesliga games [7].

Another factor influencing the frequency of COVID-19 infections among Russian soccer players could be the frequent flying, and thus a large number of close contacts. Each team had at least 20 flights, lasting >2 hours during the study period. Although the teams used charter flights, the risk of infection was nevertheless high. During teams stay at the training grounds and on the way from the bus to the “clean” zone, all the players must wear masks. But, unfortunately, not all

teams lived separately at the hotels or training grounds, in connection with which the players often stayed overnight at home. However, a higher detection rate in Russian players could be due to meticulous PCR-testing done by the physicians' staff once in a week, which allowed them to detect infections early and take actions to promptly isolate and treat the players who were tested positive.

An important aspect of the professional athlete's treatment is strict adherence to anti-doping regulations. If prohibited substance use is necessary for t an athlete has to apply for a Therapeutic Exemption (TUE) by the respective national anti-doping association. Considering that uniform COVID-19 treatment method has not been established yet, in some cases it could be problematic to receive a TUE from the WADA retroactively, e.g., after being treated (e.g. with dexamethasone [10]. However, the treatment protocols applied to soccer players did not require the use of forbidden substances such as systemic glucocorticoid use.

The recovery period length to enable the safe resumption of training is critically important for soccer players. It is assumed that COVID-19 itself as well as various therapeutics to treat the infection might cause negative effects on organs, primarily on the cardio-respiratory system [11]. However, the side effects might be sub-clinical and not appear instantly [12]. Importance of health monitoring in athletes recovered from COVID-19 was stressed in previous studies [13].

According to the Russian sports regulations, all professional athletes must undergo compulsory thorough medical examination twice in a year. Those athletes who are non-compliant to this requirement will not receive permission from national sport federation to train and participate in competitions [14]. After full recovery all soccer players who had COVID19 diagnosed passed this type of examination and none of them was diagnosed with any pulmonary or cardiac pathologies or exercise intolerance. There were no athletes who got an advice to restrain from physical activity irrespective of its intensity.

Thus, asymptomatic and symptomatic soccer players with mild pulmonary manifestations of the disease did not demonstrate any impairment in respiratory and cardiovascular function or

exercise intolerance in the short-term after recovery from COVID-19 infection. The long-term effects of COVID-19 should be the focus of future research.

Limitations of research

The research design was retrospective survey that implies possible bias (e.g., recall bias). Predominant virus strain in the time of survey was wild strain (Wuhan strain). Nowadays it is considered that new predominant strain may be more virulent and aggressive (e.g., B.1.167.2).

Conclusion

COVID-19 infection was commonly diagnosed among adult professional soccer players continuously residing in Russia. However, the majority of infections had a mild course and did not impair return to regular exercise.

What are the findings?

- COVID-19 was often diagnosed among professional soccer players continuously residing in Russia. The percentage of players who tested positive comprised 14.5%.
- Infection with COVID-19 was difficult to evade despite strict compliance with all preventive measures. Such a number of cases of covid-19 with strict sanitary regulations may indicate the indiscipline of players while at home outside matches and trainings.
- The majority of players were asymptomatic or had a mild disease course.

Perspective

- The treatment of COVID-19 in professional soccer players usually do not require the usage of drugs from the WADA List of prohibited substances.

- Symptomatic soccer players with pulmonary manifestations of the disease do not demonstrate any impairment in respiratory and cardiovascular function or exercise intolerance after recovery from COVID-19 infection. Any long-term effects are not known yet.

Competing interests: Authors declare no conflict of interest

Funding info: The author(s) received no financial support for the research, authorship, and/or publication of this article

Ethical approval information: The conduction of this study at this research institution was also approved by the local ethics committee of the Sechenov First Moscow State Medical University [extract from the Protocol 30–20 of the special meeting of the Local Ethics Committee on 21.10.2020].

Data availability:

The datasets generated and analyzed during the current study are available from the corresponding author on reasonable request.

References

1. Pedersen L, Lindberg J, Lind RR, Rasmusen H. Reopening elite sport during the COVID-19 pandemic: Experiences from a controlled return to elite football in Denmark. Scand J Med Sci Sport. 2021;31. doi:10.1111/sms.13915
2. Schumacher YO, Tabben M, Hassoun K, Al Marwani A, Al Hussein I, Coyle P, et al. Resuming professional football (soccer) during the COVID-19 pandemic in a country with high infection rates: A prospective cohort study. Br J Sports Med. 2021;55. doi:10.1136/bjsports-2020-103724

3. Chen N, Zhou M, Dong X, Qu J, Gong F, Han Y, et al. Epidemiological and clinical characteristics of 99 cases of 2019 novel coronavirus pneumonia in Wuhan, China: a descriptive study. Lancet. 2020;395. doi:10.1016/S0140-6736(20)30211-7
4. Corsini A, Bisciotti GN, Eirale C, Volpi P. Football cannot restart soon during the COVID-19 emergency! A critical perspective from the Italian experience and a call for action. British Journal of Sports Medicine. 2020. doi:10.1136/bjsports-2020-102306
5. Duarte Muñoz M, Meyer T. Infectious diseases and football–lessons not only from COVID-19. Science and Medicine in Football. 2020. doi:10.1080/24733938.2020.1749422
6. Sanitary regulations of the Russian Premier League. Season 2020/21.
7. Meyer T, Mack D, Donde K, Harzer O, Krutsch W, Rössler A, et al. Successful return to professional men's football (soccer) competition after the COVID-19 shutdown: A cohort study in the German Bundesliga. Br J Sports Med. 2021;55. doi:10.1136/bjsports-2020-103150
8. Grant MC, Geoghegan L, Arbyn M, Mohammed Z, McGuinness L, Clarke EL, et al. The prevalence of symptoms in 24,410 adults infected by the novel coronavirus (SARS-CoV-2; COVID-19): A systematic review and meta-analysis of 148 studies from 9 countries. PLoS One. 2020;15. doi:10.1371/journal.pone.0234765
9. A. B, X. M, M. H, Y. Y, Z.A. F, N. Z, et al. Chest CT Findings in Coronavirus Disease-19 (COVID-19): Relationship to Duration of Infection. Radiology. 2020;295.
10. Salvi R, Patankar P. Emerging pharmacotherapies for COVID-19. Biomedicine and Pharmacotherapy. 2020. doi:10.1016/j.biopha.2020.110267
11. Rajpal S, Tong MS, Borchers J, Zareba KM, Obarski TP, Simonetti OP, et al. Cardiovascular Magnetic Resonance Findings in Competitive Athletes Recovering from COVID-19 Infection. JAMA Cardiology. 2021. doi:10.1001/jamacardio.2020.4916
12. Guzik TJ, Mohiddin SA, Dimarco A, Patel V, Savvatis K, Marelli-Berg FM, et al.

COVID-19 and the cardiovascular system: Implications for risk assessment, diagnosis, and treatment options. Cardiovascular Research. 2020. doi:10.1093/cvr/cvaa106

13. Wilson MG, Hull JH, Rogers J, Pollock N, Dodd M, Haines J, et al. Cardiorespiratory considerations for return-to-play in elite athletes after COVID-19 infection: A practical guide for sport and exercise medicine physicians. British Journal of Sports Medicine. 2020. doi:10.1136/bjsports-2020-102710

14. Order of the Ministry of Health of the Russian Federation of March 1 2016 No. 134n. 14. Order of the Ministry of Health of the Russian Federation of March 1, 2016 No. 134n "On the Procedure for organizing the provision of medical care to persons engaged in physical culture and sports (including in the preparation and conduct of physical .

Click here to access/download;Figure;Table1.docx

Table 1. Clinical symptoms in soccer players with COVID-19 infection

**Symptom**	**Frequency in symptomatic soccer players (%, n)**
Fatigue	72.4%, 42
Headache	65.5%, 38
t ≥38 °C	44.8%, 26
(Anosmia/parosmia)	41.3%, 24
Sore throat	31%, 18
37°C<T <38 °C	27.6%, 16
Cough	15.5%, 9
Diarrhea	8.6%, 5
Myalgia	6.9%, 4
Dyspnea	1.7%, 1

Click here to access/download;Figure;Table2.docx

Table 2. Drugs utilized for the treatment of COVID-19 infection.

Drugs	Prevalence (%, n)
Antivirals (Umifenovir)	85.2%, 52
Antibiotics (Azithromycin)	83.6%, 51
Polivitamins	59%, 36
Anticoagulants (Enoxaparin)	26.2%, 16
Interferons (intranasal interferon-gamma)	14.8%, 9
Anti-malaria agents (hydroxychloroquine)	11.5%, 7
NSAIDs (COX-2 selective inhibitors)	6.6%, 4
