# Supplementary material for: The incidence and severity of COVID-19 in adult professional soccer players in Russia
Source: PLoS One. 2022 Jun 6;17(6):e0265019. doi: 10.1371/journal.pone.0265019 (PMC9170087; doi:10.1371/journal.pone.0265019)
Supplement: Attachment — Submitted filename: Point-by-point Response to Reviewers.pdf [file pone.0265019.s003.pdf]

Dear Reviewers. Great thanks for your positive approach and very valuable comments. We tried to improve the text according to your suggestions and we hope that it will satisfy you. All comments were corrected in accordance with the Reviewer's recommendations.

Reviewer #1:

This is a relevant article in the context of the pandemic, because football matches and sports were suspended for many months. Methodology is sound and design is appropriate to estimate incidence. Paper has policy implications also because it highlights the need for regular screening based on the high proportion of asymptomatic cases.

Answer: Thank you very much for your approval.

Reviewer #2:

Review summary of the article PONE-D-21-355

A. Summary of the research:

1. Major issues (according to the progress of manuscript)

♣ In the introduction part, it is necessary to mention the extent and rationale of the study in the backdrop of the objectives of the study.

Answer: Agree. We put this in the text.

♣ In the current study introduction part has been started and ended mentioning few studies in this issue which can be omitted at least at the beginning part.

Answer: Agree. Various therapeutic approaches for COVID-19 treatment are utilized, although their efficacy and safety are dubious.

♣ Objectives of the present study needed to mention at the last paragraph of the introduction.

Answer: Agree. We put the following text into the manuscript:

This research aims to investigate the clinical course of COVID-19 infection and its impact on the performance of adult professional soccer players as one of the most significant practical importance in sports medicine at the moment.

♣ In the result section, page no. 7, “admission of any of the ……….with gas analysis” to be discussed very briefly as it is supportive information to build the result but it is not studied finding directly.

Answer: Agree. We did it shorter.

Admission of any football players to participate in competitions and regular training occurs only after passing a mandatory medical examination, performed twice a year. This examination includes a routine physical examination, clinical blood and urine test, ECG (rest and stress-test), spirography, chest X-ray

♣ In the discussion part, the result of the current study will not be repeated.

Answer: Agree. Deleted.

♣ In reference part, if no. of authors >3, et al. to be mentioned.

Answer: Agree. Done.

2. Minor issues:

♣ Introductory statement of the abstract part better to describe the problem statement in brief.

Answer: Agree. We have added.

♣ Page no. 10, “according to Russian…………competition”, the relevance of this statement to the current study is not clear.

Answer: The presence of a mandatory additional examination before admitting an athlete to physical and sports activity will allow identifying various deviations in the health status of athletes after suffering from COVID.

♣ The 2nd limitation, “predominant virus strain………” is questionable/needs clarity as a study limitation.

Answer: Agree. Sure, it is not a limitation of the research. But this aspect must be taken into account when reading the article since all the results were obtained in a specific period of time when a specific strain of the virus prevailed.
